# Peripheral T cell cytotoxicity predicts the efficacy of anti-PD-1 therapy for advanced non-small cell lung cancer patients

**DOI:** 10.1038/s41598-022-22356-0

**Published:** 2022-10-19

**Authors:** Kota Iwahori, Takeshi Uenami, Yukihiro Yano, Toshihiko Ueda, Mari Tone, Yujiro Naito, Yasuhiko Suga, Kiyoharu Fukushima, Takayuki Shiroyama, Kotaro Miyake, Shohei Koyama, Haruhiko Hirata, Izumi Nagatomo, Hiroshi Kida, Masahide Mori, Yoshito Takeda, Atsushi Kumanogoh, Hisashi Wada

**Affiliations:** 1 Department of Clinical Research in Tumor Immunology, Graduate School of Medicine, Osaka University, 2-2 Yamadaoka, Suita, Osaka 565-0871 Japan; 2 Department of Respiratory Medicine and Clinical Immunology, Graduate School of Medicine, Osaka University, Osaka, Japan; 3 Department of Thoracic Oncology, National Hospital Organization Osaka Toneyama Medical Center, Osaka, Japan; 4 Bioanalysis Department, Advanced Technology Center, Medical Solution Segment, LSI Medience Corporation, Tokyo, Japan; 5 Department of Respiratory Medicine, National Hospital Organization Osaka Toneyama Medical Center, Osaka, Japan; 6 Department of Immunopathology, Immunology Frontier Research Center, Osaka University, Osaka, Japan; 7 Integrated Frontier Research for Medical Science Division, Institute for Open and Transdisciplinary Research Initiatives (OTRI), Osaka University, Osaka, Japan

**Keywords:** Cancer, Immunology, Biomarkers, Oncology

## Abstract

Anti-programmed cell death-1 (PD-1) therapy exerts beneficial effects in a limited population of cancer patients. Therefore, more accurate diagnostics to predict the efficacy of anti-PD-1 therapy are desired. The present study investigated whether peripheral T cell cytotoxicity predicts the efficacy of anti-PD-1 therapy for advanced non-small cell lung cancer (NSCLC) patients. Advanced NSCLC patients treated with anti-PD-1 monotherapy (nivolumab or pembrolizumab) were consecutively enrolled in the present study. Peripheral blood samples were subjected to an analysis of peripheral T cell cytotoxicity and flow cytometry prior to the initiation of anti-PD-1 therapy. Peripheral T cell cytotoxicity was assessed using bispecific T-cell engager (BiTE) technology. We found that progression-free survival was significantly longer in patients with high peripheral T cell cytotoxicity (*p* = 0.0094). In the multivariate analysis, treatment line and peripheral T cell cytotoxicity were independent prognostic factors for progression-free survival. The analysis of T cell profiles revealed that peripheral T cell cytotoxicity correlated with the ratio of the effector memory population in CD4+ or CD8+ T cells. Furthermore, the results of flow cytometry showed that the peripheral CD45RA+CD25+/CD4+ T cell ratio was higher in patients with than in those without severe adverse events (*p* = 0.0076). These results indicated that the peripheral T cell cytotoxicity predicted the efficacy of anti-PD-1 therapy for advanced NSCLC patients.

## Introduction

Anti-programmed cell death-1 (PD-1) therapy exerts beneficial effects in a limited population of cancer patients. PD-Ligand 1 (PD-L1) staining has been developed for companion diagnostics to this treatment. The KEYNOTE-024 trial indicated that pembrolizumab was associated with significantly longer progression-free survival (PFS) and overall survival (OS) than platinum-based chemotherapy in patients with advanced non-small cell lung cancer (NSCLC) and PD-L1 expressed on at least 50% of tumor cells^1^. Recently, based on the KEYNOTE-042 trial, the Food and Drug Administration (FDA) approved pembrolizumab as the first-line treatment for patients with advanced NSCLC expressing PD-L1 on at least 1% of tumor cells without EGFR or ALK genomic aberrations^2^. The expanded application of pembrolizumab to patients with a PD-L1 tumor proportion score (TPS) ≥ 1% increased the number of patients eligible for anti-PD-1 therapy instead of reducing the proportion of responders to this treatment. Other than PD-L1 staining, biomarkers that predict the efficacy of PD-1 inhibitors are currently limited. More accurate diagnostics for predicting the efficacy of anti-PD-1 therapy are desired.

Some patients who receive anti-PD-1 therapy develop adverse events (AEs). NSCLC patients who developed immune-related adverse events (irAEs) from anti-PD-1 therapy achieved better outcomes than those without irAEs^3–8^. However, recent studies indicated that among NSCLC patients with irAEs, OS was slightly shorter in patients with than in those without severe irAEs^9–11^. Therefore, in addition to companion diagnostics for treatment efficacy, there is a growing need for biomarkers that predict severe irAEs in order to achieve better clinical outcomes from anti-PD-1 therapy.

The critical point of action for cancer immunotherapy including anti-PD-1 therapy is tumor antigen-specific T cell cytotoxicity to tumor cells in the tumor microenvironment. A previous study detected tumor antigen-specific T cells in the peripheral blood of cancer patients^12^. Furthermore, T cell clones were shared between tumor tissue and peripheral blood in metastatic melanoma patients^13–17^. We developed an assay system for T cell cytotoxicity using bispecific T cell engager (BiTE) technology and reported that peripheral T cell cytotoxicity correlated with T cell cytotoxicity in the tumor microenvironment of NSCLC patients^18,19^. Based on these findings, we hypothesized that peripheral T cell cytotoxicity may predict the efficacy of anti-PD-1 therapy.

Therefore, the present study investigated whether peripheral T cell cytotoxicity predicts the efficacy of anti-PD-1 therapy for advanced NSCLC patients. We also searched for biomarkers of severe AEs associated with anti-PD-1 therapy.

## Results

### Peripheral T cell cytotoxicity correlates with the efficacy of anti-PD-1 therapy

**Figure 1:**
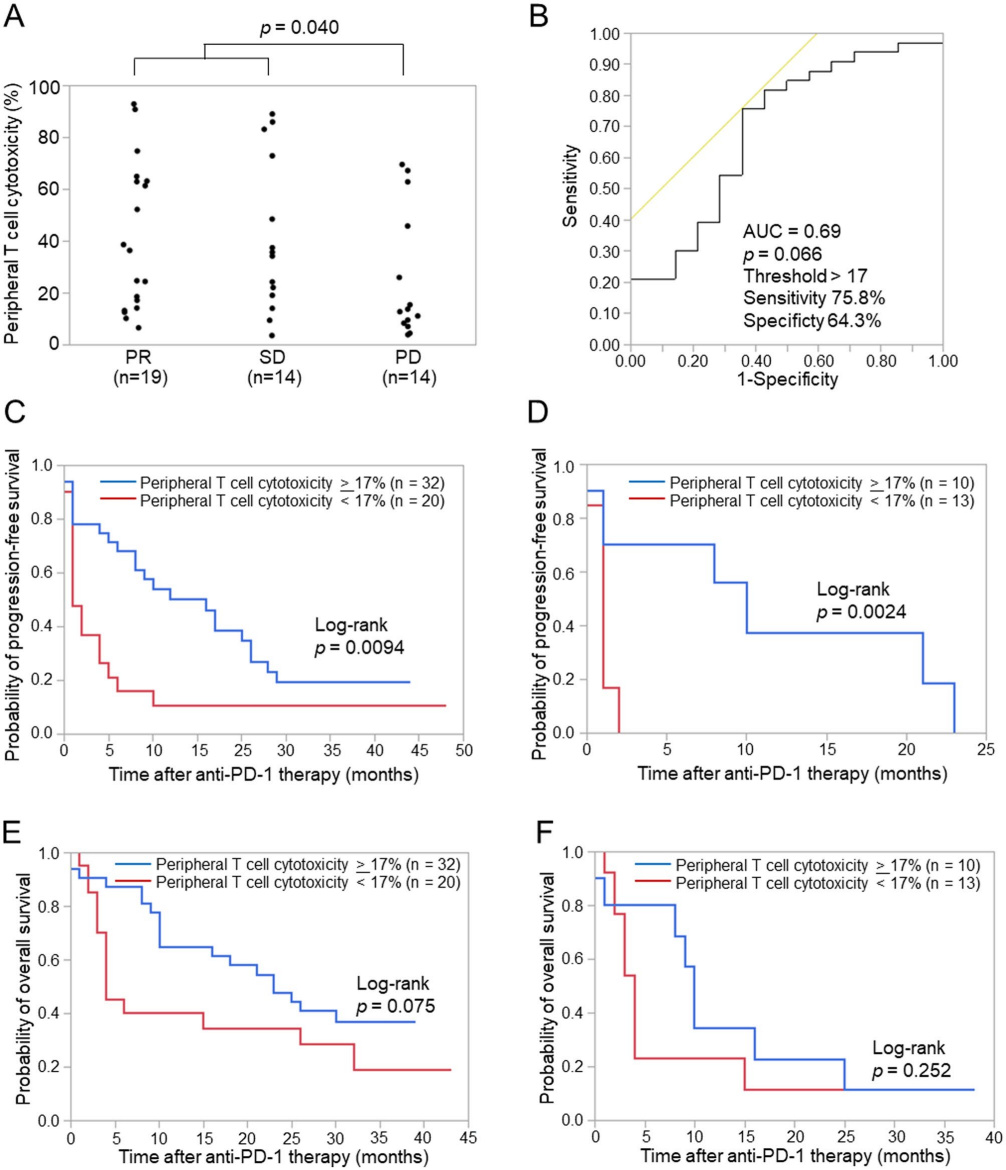
Relationship between peripheral T cell cytotoxicity and clinical responses to anti-PD-1 therapy. (**A**) Peripheral T cell cytotoxicity according to objective tumor responses to anti–PD-1 therapy. Peripheral T cell cytotoxicity was measured prior to the initiation of anti-PD-1 therapy. Responses were evaluated according to the Response Evaluation Criteria in Solid Tumours (RECIST) version 1.1 guidelines as a partial response (PR), stable disease (SD), or progressive disease (PD). Each dot represents one patient. The significance of differences was assessed using the Mann–Whitney U test. (**B**) Receiver operating characteristic (ROC) curves for peripheral T cell cytotoxicity to differentiate patients with PR and SD from those with PD. (**C**) Kaplan–Meier curves for the progression-free survival (PFS) of patients for the first-line or later treatments according to peripheral T cell cytotoxicity levels. (**D**) Kaplan–Meier curves for the PFS of patients for the second-line or later treatments according to peripheral T cell cytotoxicity levels. (**E**) Kaplan–Meier curves for the overall survival (OS) of patients for the first-line or later treatments according to peripheral T cell cytotoxicity levels. (**F**) Kaplan–Meier curves for the OS of patients for the second-line or later treatments according to peripheral T cell cytotoxicity levels. The significance of differences was assessed using the log-rank test (**C**–**F**). AUC, area under the curve.

Fifty-two patients with NSCLC who were treated with nivolumab or pembrolizumab were enrolled (Table 1), and peripheral T cell cytotoxicity and flow cytometry were performed using pre-treated peripheral blood. Peripheral blood samples from 52 patients were subjected to peripheral T cell cytotoxicity and those from 43 patients to flow cytometry prior to anti-PD-1 therapy (Supplementary Fig. S1). The median follow-up time after anti-PD-1 therapy was 399 days (range, 18–1310 days). Regarding objective responses, peripheral T cell cytotoxicity was significantly higher in patients with a partial response (PR) and stable disease (SD) than in those with progressive disease (PD) (Fig. 1A, *p* = 0.040). Patients who were not evaluable (NE, n = 5) included those who discontinued anti-PD-1 therapy and in whom an objective response was not evaluable due to a decreased performance status. To assess the ability of peripheral T cell cytotoxicity to predict responses to anti-PD-1 therapy, we examined the area under the receiver operating characteristic (ROC) curve (AUC) for peripheral T cell cytotoxicity. The AUC was 0.69 for differentiating PR and SD (*n* = 33) from PD (*n* = 14) for the effects of anti-PD-1 therapy with a cut-off value of 17% (sensitivity = 75.8%, specificity = 64.3%, *p* = 0.066) (Fig. 1B).

**Table 1:** Patient characteristics.

Patient characteristics	Overall
	(n = 52)
**Age, years**	
Median	71
Range	40–82
**Sex, n (%)**	
Male	36 (69.2)
Female	16 (30.8)
**Smoking status, n (%)**	
Current or former smoker	47 (90.4)
Never smoked	5 (9.6)
**Histology, n (%)**	
Squamous	14 (26.9)
Non-squamous	38 (73.1)
**Treatment line, n (%)**	
1st line	29 (55.8)
≥ 2nd line or later	23 (44.2)
**PD-L1 expression level, n (%)**	
≥ 50%	34 (65.4)
< 50%	17 (32.7)
Unknown	1 (1.9)

**Figure 2:**
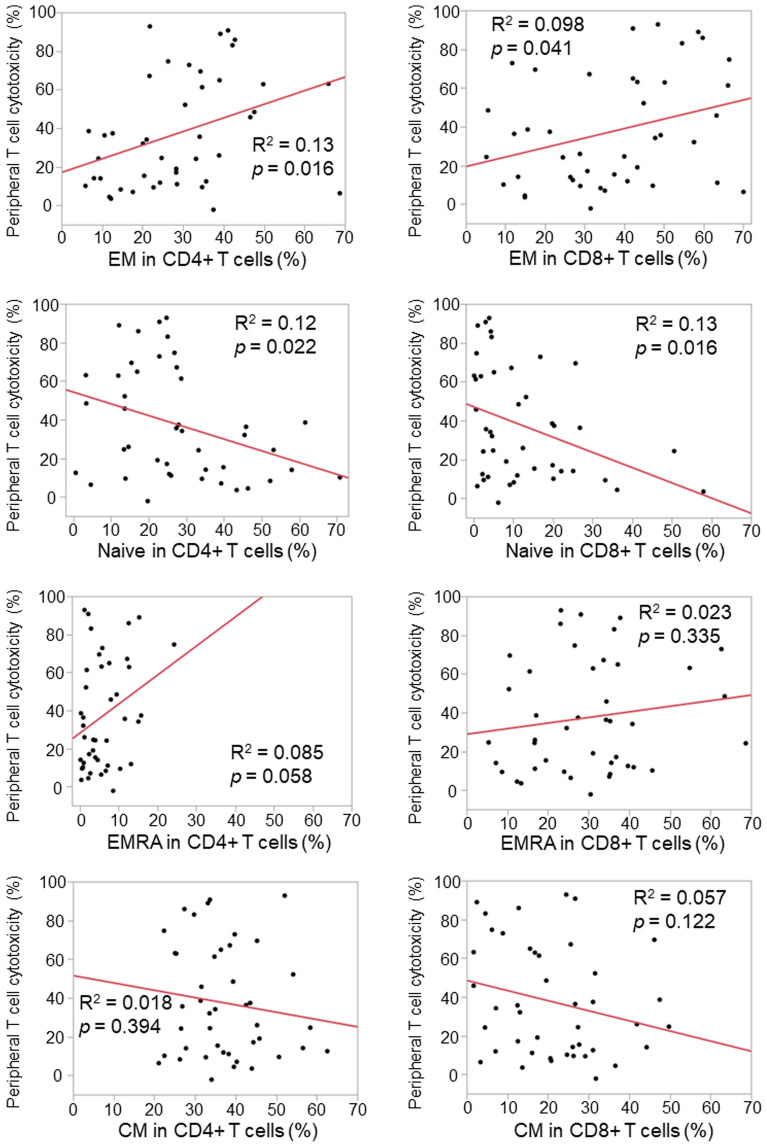
Correlation between peripheral T cell cytotoxicity and the peripheral T cell profile. The ratios of naïve (CD45RA+CD27+), central memory (CM, CD45RA-CD27+), effector memory (EM, CD45RA−CD27−), and effector memory re-expressing CD45RA (EMRA, CD45RA+CD27−) populations in CD4+ and CD8+ T cells were analyzed for correlations with peripheral T cell cytotoxicity. Each dot represents one patient (n = 43). Correlations between paired data were analyzed using Pearson’s correlation coefficient.

Based on the cut-off value of 17% for peripheral T cell cytotoxicity, we analyzed PFS and OS in patients with high peripheral T cell cytotoxicity (peripheral T cell cytotoxicity ≥ 17%) and low peripheral T cell cytotoxicity (peripheral T cell cytotoxicity < 17%). PFS was significantly longer in patients with high peripheral T cell cytotoxicity than in those with low peripheral T cell cytotoxicity in a cohort of patients after the first-line treatments (Fig. 1C, *p* = 0.0094) as well as after the second-line treatments (Fig. 1D, *p* = 0.0024). OS was slightly longer in patients with high peripheral T cell cytotoxicity than in those with low peripheral T cell cytotoxicity after the first-line treatments (*p* = 0.075) as well as after the second-line treatments (*p* = 0.252) (Fig. 1E,F). The multivariate analysis revealed that histology (HR = 0.44, 95% CI: 0.20–0.98, *p* = 0.045), the treatment line (HR = 0.24, 95% CI: 0.08–0.68, *p* = 0.0071), and peripheral T cell cytotoxicity (HR = 0.33, 95% CI: 0.15–0.72, *p* = 0.0058) were independent prognostic factors for PFS, while age (HR = 0.43, 95% CI: 0.19–0.97, *p* = 0.041), the treatment line (HR = 0.31, 95% CI: 0.10–0.96, *p* = 0.042), and PD-L1 expression levels (HR = 0.26, 95% CI: 0.09–0.73, *p* = 0.011) were independent prognostic factors for OS (Table 2). In terms of PD-L1 expression levels, 93% (27/29) of patients with high PD-L1 expression (≥ 50%) received anti-PD-1 therapy as the first-line treatment. On the other hand, significantly fewer patients (7/22, 32%) with low PD-L1 expression (< 50%) received anti-PD-1 therapy as the first-line treatment (χ^2^ test, *p* < 0.0001). No significant difference was observed in peripheral T cell cytotoxicity between patients with high PD-L1 expression (≥ 50%) and low PD-L1 expression (< 50%) (Supplementary Fig. S2). To elucidate the relationship between peripheral T cell cytotoxicity and the T cell profile, we examined flow cytometric data on the peripheral blood of each patient. The analysis of T cell profiles revealed that peripheral T cell cytotoxicity correlated with the ratio of the effector memory population (CD45RA-CD25-) in CD4+ or CD8+ T cells (Fig. 2). Peripheral T cell cytotoxicity was decreased by CD4+ or CD8+ T cell depletion (Supplementary Fig. S3). Taken together with these results, peripheral T cell cytotoxicity reflects the cytotoxic activity of effector memory T cells and has the potential to predict the efficacy of anti-PD-1 therapy for NSCLC patients.

**Table 2:** Univariate and multivariate analyses of progression-free survival (PFS) and overall survival (OS).

	Progression free survival						Overall survival					
	Univariate analysis			Multivariate analysis			Univariate analysis			Multivariate analysis		
	HR	95% CI	*p value*	HR	95% CI	*p value*	HR	95% CI	*p value*	HR	95% CI	*p value*
Age (< 70 vs. ≥ 70)	1.01	0.54–1.89	0.96	0.9	0.44–1.82	0.77	0.86	0.43–1.69	0.65	0.43	0.19–0.97	0.041
Sex (female vs. male)	0.88	0.44–1.73	0.7	0.99	0.43–2.27	0.97	0.83	0.40–1.74	0.62	0.52	0.21–1.29	0.16
Smoking status (current/former vs. never)	1.13	0.40–3.19	0.82	0.72	0.19–2.70	0.63	1.46	0.45–4.79	0.53	0.63	0.16–2.44	0.5
Histology (non-Sq vs. Sq)	0.84	0.42–1.65	0.61	0.44	0.20–0.98	0.045	1.18	0.53–2.62	0.68	0.68	0.27–1.73	0.42
Treatment line (1st vs. ≥ 2nd)	0.33	0.17–0.62	0.0007	0.24	0.08–0.68	0.0071	0.27	0.13–0.54	0.0002	0.31	0.10–0.96	0.042
PD-L1 expression level (≥ 50% vs. < 50%)	0.42	0.21–0.84	0.014	0.93	0.35–2.47	0.35	0.24	0.12–0.48	< 0.0001	0.26	0.09–0.73	0.011
Peripheral T cell cytotoxicity (≥ 17% vs. < 17%)	0.42	0.22–0.80	0.0086	0.33	0.15–0.72	0.0058	0.55	0.28–1.08	0.084	0.56	0.26–1.22	0.15

Univariate and multivariate analyses were performed using the Cox proportional hazards regression to evaluate the relationships of all factors with PFS and OS.

HR, Hazard ratio; CI, confidence interval.

### The CD45RA+CD25+/CD4+ T cell ratio correlates with severe AEs of anti-PD-1 therapy

**Figure 3:**
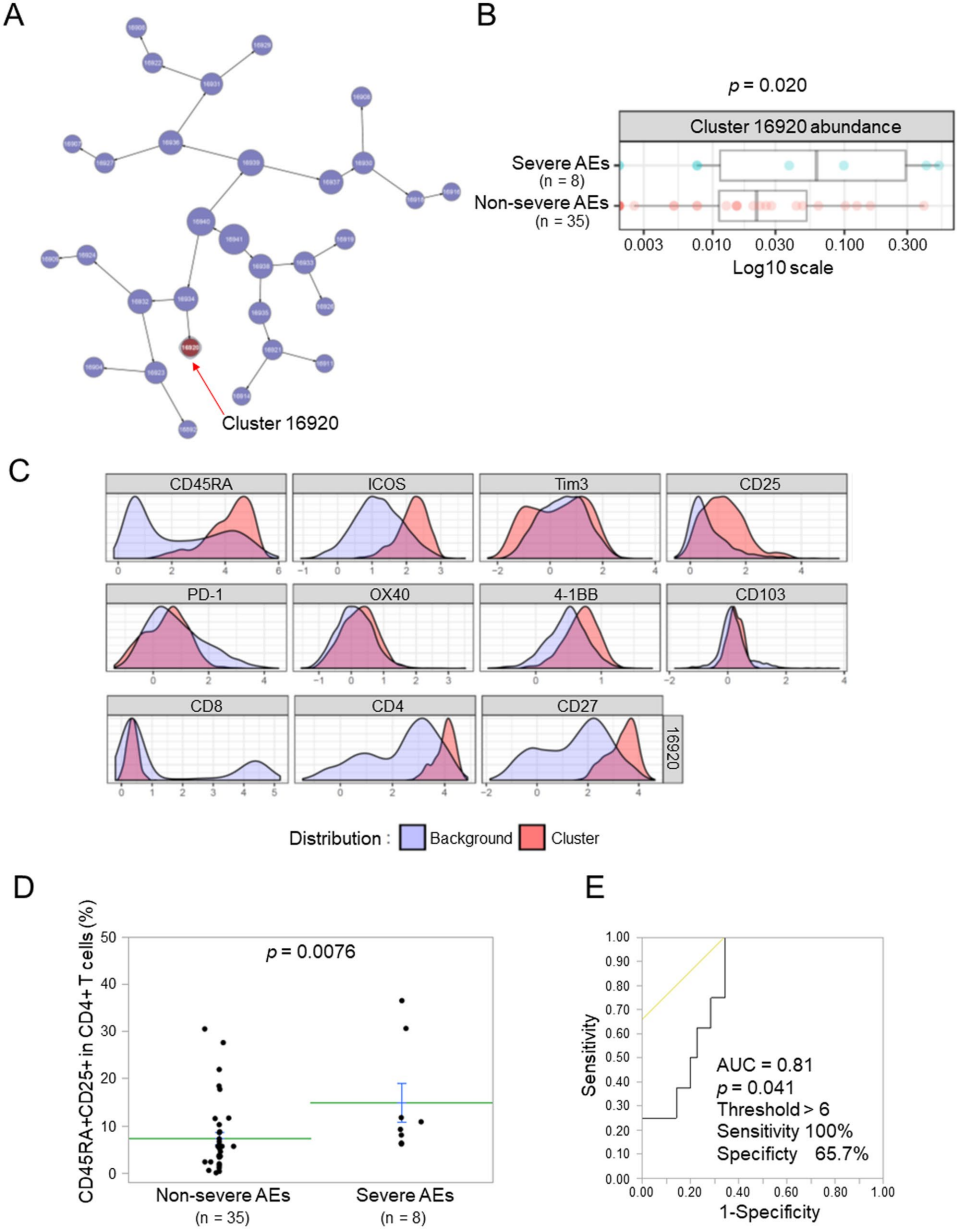
Relationship between the peripheral T cell profile and severe adverse events (AEs). (**A**) A CITRUS analysis of pre-treatment samples comparing severe AEs (n = 8) versus non-severe AEs (n = 35). The abundance of the cluster significantly differed between the groups, as shown in the CITRUS cluster tree. (**B**) The abundance summary plot of the cluster that was significantly different between severe AEs and non-severe AEs. (**C**) Phenotype histograms of the cluster that increased in patients with severe AEs. (**D**) A comparison of the CD45RA+CD25+/CD4+ T cell ratio between severe AEs (n = 8) and non-severe AEs (n = 35). Each dot represents one patient. Data represent the mean ± standard error of the mean (SEM). The significance of differences was assessed using the Mann–Whitney's U test. (**E**) Receiver operating characteristic (ROC) curves for the CD45RA+CD25+/CD4+ T cell ratio to differentiate patients with severe AEs from those with non-severe AEs. AE, adverse event; AUC, area under the curve.

Among the 52 patients in the present study, nine developed severe AEs that led to the discontinuation of anti-PD-1 therapy (Supplementary Table 1). The most common severe AE was pneumonitis (*n* = 3). Among them, one patient with pneumonitis had treatment-related death. The median time for the duration of anti-PD-1 therapy with severe AEs was 25 days. To identify the factors contributing to severe AEs, we compared T cell profiles between patients with and without severe AEs using the CITRUS (cluster identification, characterization, and regression) analysis with Cytobank software. CITRUS identified a cluster of T cells in peripheral blood that was more abundant in patients with than in those without severe AEs (Fig. 3A,B). Surface marker expression indicated that this cluster included CD4+CD45RA+CD25+ T cells (Fig. 3C). The results of flow cytometry showed that the CD45RA+CD25+/CD4+ T cell ratio was significantly higher in patients with than in those without severe AEs (Fig. 3D and Supplementary Fig. S4, *p* = 0.0076). Intracellular Foxp3 staining revealed that the majority of CD4+CD45RA+CD25+ T cells did not express Foxp3 (Supplementary Fig. S5). To assess the predictive value of the CD45RA+CD25+/CD4+ T cell ratio for severe AEs, we examined the AUC for the CD45RA+CD25+/CD4+ T cell ratio. The AUC was 0.81 for differentiating severe AEs (*n* = 8) from non-severe AEs (*n* = 35) with a cut-off value of 6% (sensitivity = 100%, specificity = 65.7%, *p* = 0.041) (Fig. 3E).

**Figure 4:**
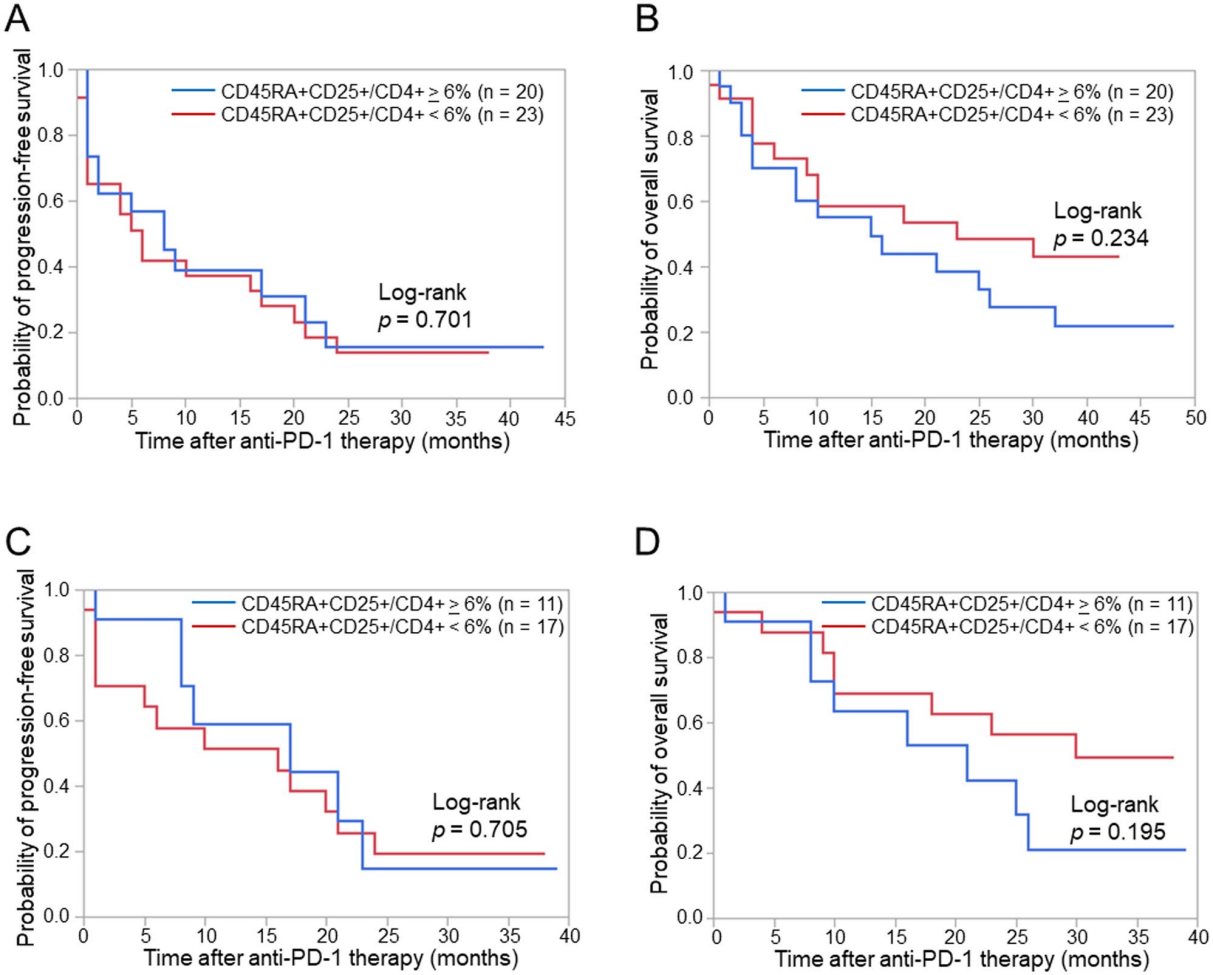
Progression-free survival (PFS) and overall survival (OS) according to the combination of peripheral T cell cytotoxicity and the peripheral CD45RA+CD25+/CD4+ T cell ratio. (**A**) Kaplan–Meier curves for the PFS of patients according to the CD45RA+CD25+/CD4+ T cell ratio. (**B**) Kaplan–Meier curves for the OS of patients according to the CD45RA+CD25+/CD4+ T cell ratio. (**C**) Kaplan–Meier curves for the PFS of patients with peripheral T cell cytotoxicity ≥ 17% according to the CD45RA+CD25+/CD4+ T cell ratio. (**D**) Kaplan–Meier curves for the OS of patients with peripheral T cell cytotoxicity ≥ 17% according to the CD45RA+CD25+/CD4+ T cell ratio. The significance of differences was assessed using the Log-rank test (**A**–**D**).

Based on the cut-off value of 6% for the CD45RA+CD25+/CD4+ T cell ratio, we assessed PFS and OS in patients with a high CD45RA+CD25+/CD4+ T cell ratio (≥ 6%) and in those with a low CD45RA+CD25+/CD4+ T cell ratio (< 6%). Although no significant differences were observed in PFS between patients with high and low CD45RA+CD25+/CD4+ T cell ratios, OS was slightly longer in patients with a low CD45RA+CD25+/CD4+ T cell ratio (Fig. 4A,B). To distinguish subgroups of patients for anti-PD-1 therapy, we verified the combination of peripheral T cell cytotoxicity and the CD45RA+CD25+/CD4+ T cell ratio. Regarding PFS, no significant difference was noted between patients with high and low CD45RA+CD25+/CD4+ T cell ratios in the high peripheral T cell cytotoxicity group. In contrast, OS was slightly longer in patients with a low CD45RA+CD25+/CD4+ T cell ratio than in those with a high CD45RA+CD25+/CD4+ T cell ratio (Fig. 4C,D). Similar results were obtained for patients with high PD-L1 expression (≥ 50%) (Supplementary Fig. S6). These results indicated that patients with high peripheral T cell cytotoxicity and a low CD45RA+CD25+/CD4+ T cell ratio achieved good OS without severe AEs, while those with high peripheral T cell cytotoxicity and a high CD45RA+CD25+/CD4+ T cell ratio had inferior OS and developed severe AEs despite similar PFS. Taken together with these results, the combination of peripheral T cell cytotoxicity and the peripheral CD45RA+CD25+/CD4+ T cell ratio predicted the efficacy and severe adverse events of anti-PD-1 therapy for advanced NSCLC patients.

## Discussion

The present results demonstrated that peripheral T cell cytotoxicity predicted the efficacy of anti-PD-1 therapy for advanced NSCLC patients. Furthermore, the peripheral CD45RA+CD25+/CD4+ T cell ratio was higher in patients with than in those without severe AEs. The combination of peripheral T cell cytotoxicity and the peripheral CD45RA+CD25+/CD4+ T cell ratio distinguishes patients with severe AEs from those with non-severe AEs among responders to anti-PD-1 therapy. These approaches have highlighted the need for novel strategies to prevent severe AEs in responders, leading to better treatment outcomes with anti-PD-1 therapy.

We developed peripheral T cell cytotoxicity analysis as a biomarker of anti-PD-1 therapy based on our previous findings on the relationship between T cell cytotoxicity in the peripheral blood and tumor tissue of NSCLC patients^18^. It shows the sum of cytotoxicity for individual peripheral T cells because a fixed number of peripheral blood mononuclear cells (PBMCs) is used in this assay. Specifically, peripheral T cell cytotoxicity reflects the cytotoxicity of effector memory T cells, which expand intratumorally and peripherally in response to immune checkpoint inhibitors (ICIs)^15,20,21^. Previous studies indicated that peripheral T cell functions correlated with PFS with ICIs^14,22,23^.

In the present study, the CD45RA+CD25+/CD4+ T cell ratio was higher in patients with than in those without severe AEs. We defined severe AEs as AEs leading to the discontinuation of anti-PD-1 therapy. irAEs were defined as AEs related to ICIs and not caused by disease progression or other factors. The majority of severe AEs in the present study were irAEs; however, some were not pathologically identified as irAEs. CD4+CD45RA+CD25+ T cells include naïve regulatory T (Treg) cells expressing CD45RA as a marker of naïve T cells^24,25^. However, CD4+CD45RA+CD25+ T cells were considered to consist of not only naïve Treg cells, but also activated CD4+ T cells expressing CD25 as an activation marker. Furthermore, the definition of markers for Treg subsets differs among studies. Therefore, the interpretation of the relationship between severe AEs and CD4+CD45RA+CD25+ T cells needs to consider naïve Treg and activated CD4+ T cells. The frequency of irAEs was shown to be higher in patients with a lower proportion of effector Treg cells before anti-PD-1 therapy^26^. However, the relationship between naïve Treg cells and irAEs currently remains unclear. We examined the CD45RA+CD25+/CD4+ T cell ratio before, but not after anti-PD-1 therapy. A previous study on the peripheral T cell profiles of NSCLC patients before and early after anti-PD-1 therapy indicated that patients with severe irAEs (defined as grade 3 and higher) exhibited significantly lower fold increases in the frequency of effector Treg cells^27^. The significance of monitoring the CD45RA+CD25+/CD4+ T cell ratio during anti-PD-1 therapy needs to be elucidated. On the other hand, a higher percentage of activated peripheral CD4+ T cells was detected in patients with irAEs before the second treatment cycle of ICIs^28^. The frequency of peripheral cytotoxic CD4+ T cells was shown to correlate with destructive thyroiditis induced by anti-PD-1 therapy^29^. A recent study on T cell characteristics with severe irAEs (defined as grade 3 and higher) revealed that the abundance of activated peripheral CD4+ memory T cells before ICIs was associated with the development of severe irAEs in melanoma patients^30^.

Previous studies examined irAEs and the prognosis of cancer patients treated with ICIs. The prognosis of NSCLC patients with irAEs was better than that of patients without irAEs^3–8^. However, discrepancies were noted in the prognosis of patients with severe irAEs between studies^9–11,31–34^. OS was considered to be worse in patients with than in those without severe irAEs because of the discontinuation of ICIs, permanent tissue damage, or even fatal outcomes due to severe irAEs. In the present study, OS was slightly shorter in patients with a high CD45RA+CD25+/CD4+ T cell ratio than in those with a low CD45RA+CD25+/CD4+ T cell ratio, whereas PFS was similar. This result was attributed to the correlation between a high CD45RA+CD25+/CD4+ T cell ratio and severe AEs related to treatment discontinuation and tissue damage, leading to the inferior OS despite similar PFS, which is closely related to tumor regression by anti-PD-1 therapy.

There are several limitations that need to be addressed. The sample size was small without a validation cohort specifically for the analysis of severe AEs. Further studies are needed to show that ORR, PFS, and OS differ using the same threshold in an independent validation cohort. We are planning a future study to validate the present results in another independent cohort. Furthermore, severe AEs consisted of heterogeneous events that need a large sample size for the validation of each type of severe AE. Although we found that the majority of CD4+CD45RA+CD25+ T cells in patients with severe AEs did not express Foxp3, the further characterization of CD45RA+CD25+CD4+ T cells is needed. Moreover, due to the previous application of first-line anti-PD-1 monotherapy for TPS ≥ 50%, there was bias between first-line and later treatment groups for PD-L1 expression levels on tumor cells.

In conclusion, the combination of peripheral T cell cytotoxicity and the peripheral CD45RA+CD25+/CD4+ T cell ratio predicted the efficacy and severe AEs of anti-PD-1 therapy for advanced NSCLC patients. A further large-scale verification is required to establish novel strategies for anti-PD-1 therapy.

## Methods

### Study patients

Advanced NSCLC patients with metastatic/recurrent/unresectable stages or postoperative recurrence were consecutively enrolled in the present study from Osaka University Hospital and Osaka Toneyama Medical Center between November 2017 and August 2019. This is an observational study on practical treatment. Patients were treated with anti-PD-1 monotherapy (nivolumab or pembrolizumab) in the first-line or later settings. Patients with EGFR mutations or ALK translocations were excluded from the analysis because they cannot be enrolled in future front-line cancer immunotherapy trials. The peripheral blood of the enrolled patients was subjected to a peripheral T cell cytotoxicity analysis and flow cytometry prior to the initiation of anti-PD-1 therapy. Tumor PD-L1 expression was evaluated by immunohistochemistry using the 22C3 antibody at each institute. The cut-off date for data collection was June 30, 2021.

Objective responses were evaluated according to the Response Evaluation Criteria in Solid Tumours (RECIST) version 1.1. PFS was measured from the date of the initiation of anti-PD-1 therapy to the date of disease progression according to RECIST version 1.1. PFS without disease progression was censored at the date of last known contact. OS was measured from the start of anti-PD-1 therapy to death. OS for living patients was censored at the date of last known contact. Severe AEs were defined as AEs leading to the discontinuation of anti-PD-1 therapy.

The study protocol was approved by the Institutional Ethics Committee of Osaka University Hospital and Osaka Toneyama Medical Center, and written informed consent was obtained from participants prior to their inclusion in the study. The present study was conducted according to the principles of the Declaration of Helsinki.

### Construction of BiTE

We previously described the construction of BiTE containing EphA2-specific scFv 4H5, a short serine-glycine linker, and CD3-specific scFv derived from OKT3^18,35^. Specifically, the EphA2-specific engager consists of a 4H5 heavy-chain, glycine (G) serine (S) linker [(G4S)3], 4H5 light-chain, short G4S linker, OKT3 heavy-chain, (G4S)3 linker, and OKT3 light-chain. A 6 × His-Myc tag was inserted at the C terminus before the stop codon. This recombinant protein was custom-made by Thermo Fisher Scientific. Briefly, EphA2-specific engager DNA was synthesized and subcloned into the pcDNA3.3 vector. The plasmid vector was transfected into Expi293™ cells. After the culturing of cells, the protein was purified by His-tag affinity chromatography from the culture supernatant.

### Peripheral T cell cytotoxicity analysis

U251 cells that express EphA2 on their surface were kindly provided by Dr. Yasuko Mori (Kobe University, Japan). Cell line authentication by short tandem repeat profiling and mycoplasma testing were performed by the JCRB Cell Bank. U251 cells were plated on 96-well flat-bottomed cell culture plates (Corning) at a density of 1 × 10^4^ cells per well with PRMI medium 1640 (Nacalai Tesque) containing 10% fetal bovine serum (FBS; HyClone, Thermo Scientific). Cells were cultured at 37 °C in a humidified atmosphere with 5% CO_2_. After 24 h, 5 × 10^4^ freshly isolated PBMCs were added to plates with 100 ng/ml of EphA2/CD3 BiTE. PBMCs were isolated from peripheral blood by gradient density centrifugation using Lymphoprep (Axis Shield). After a 48-h co-culture, non-adherent cells were removed by gentle washing four times with RPMI medium 1640 containing 10% FBS, and the remaining adherent viable cells were detected using the 3-(4,5-dimethylthiazol-2-yl)-5-(3-carboxymethoxyphenyl)-2-(4-sulfophenyl)-2H-tetrazolium assay (CellTiter 96 aqueous one solution cell proliferation assay; Promega). The assay was performed in triplicate. The calculation of EphA2/CD3 BiTE-mediated peripheral T cell cytotoxicity against U251 cells was based on the extent of the decrease in viable target U251 cells using the following formula:

$$\% {\text{peripheral T cell cytotoxicity}} = \left[ {1 - \left( {\text{absorbance of treated wells}} \right)/\left( {\text{absorbance of non - treated wells}} \right)} \right] \times 100$$

Each treated well consisted of 1 × 10^4^ U251 cells and 5 × 10^4^ PBMCs with 100 ng/ml of EphA2/CD3 BiTE. Each non-treated well consisted of 1 × 10^4^ U251 cells and 5 × 10^4^ PBMCs without EphA2/CD3 BiTE.

To validate the reproducibility of the peripheral T cell cytotoxicity analysis, we compared the results of the peripheral T cell cytotoxicity assays of five donors measured at the laboratories of Osaka University and LSI Medience Corporation. Fresh PBMCs were analyzed at the laboratory of Osaka University and the same PBMCs were analyzed at the laboratory of LSI Medience Corporation on the next day after transportation from Osaka to Tokyo at room temperature. The results of peripheral T cell cytotoxicity assays performed at Osaka University were consistent with those at LSI Medience Corporation (Supplementary Fig. S7).

### Flow cytometry

White blood cells were extracted from peripheral blood by lysis with BD Pharm Lyse (BD Biosciences) red blood cell lysis buffer and then subjected to flow cytometry. Surface marker staining was performed after the FcR block using Human TruStain FcX Fc Receptor blocking solution (BioLegend). Surface marker-stained cells were analyzed on BD LSRFortessa with FACSDiva software (BD Biosciences). The gating strategy of the FACS analysis is shown in Supplementary Fig. S8. The following antibodies were used for FACS staining: anti-CD45RA-FITC (clone HI100), anti-CD25-PE (BC96), anti-4-1BB-BV421 (4B4-1), anti-CD8-BV510 (RPA-T8), anti-CD103-BV605 (Ber-ACT8), anti-CD4-BV711 (OKT4), anti-Tim-3-APC (F38-2E2), anti-CD3-Alexa Fluor 700 (UCHT1), and IgG1 isotype control (MOPC-21) purchased from Biolegend. Anti-ICOS-PerCP-eFluor 710 (ISA-3) and IgG1 isotype control (P3.6.2.8.1) were purchased from eBioscience. Anti-OX-40-PE-CF594 (ACT35), anti-CD27-BV786 (L128), and anti-PD-1-PE Cy7 (EH12.1) were purchased from BD Bioscience. Anti-CD19-APC-eFluor 780 (H1B19) was purchased from Invitrogen.

### Statistical analysis

The Mann–Whitney U test was used to examine the significance of differences between samples. Relationships between paired data were analyzed using Pearson’s correlation coefficient. The cut-off value was calculated by a receiver operating characteristic analysis. PFS and OS were estimated using the Kaplan–Meier method. Differences in PFS and OS between patient subgroups were examined using the log-rank test. Univariate and multivariate Cox proportional hazards models were used to estimate hazard ratios (HR) with 95% confidence intervals (CI).

A *P* value < 0.05 was considered to be significant in all tests. All analyses were performed with JMP Pro 15 software (SAS Institute, Inc.).

## Supplementary Information

Supplementary Information.

## Data Availability

The authors declare that the main data supporting the results of the present study are available within the article and its Supplementary Information files. Extra data are available from the corresponding author upon request.
