# Supplementary material for: Peripheral T cell cytotoxicity predicts the efficacy of anti-PD-1 therapy for advanced non-small cell lung cancer patients
Source: Sci Rep. 2022 Oct 19;12:17461. doi: 10.1038/s41598-022-22356-0 (PMC9582215; doi:10.1038/s41598-022-22356-0)
Supplement: Supplementary file 1 — Supplementary Information. [file 41598_2022_22356_MOESM1_ESM.pdf]

Peripheral T cell cytotoxicity predict the efficacy of anti-PD-1 therapy for advanced non-small cell lung cancer patients

Kota Iwahori,[1,2,*] Takeshi Uenami,[3] Yukihiro Yano,[3] Toshihiko Ueda,[4] Mari Tone,[1,2] Yujiro Naito,[2] Yasuhiko Suga,[2] Kiyoharu Fukushima,[2] Takayuki Shiroyama,[2] Kotaro Miyake,[2] Shohei Koyama,[2] Haruhiko Hirata,[2] Izumi Nagatomo,[2] Hiroshi Kida,[5] Masahide Mori,[3] Yoshito Takeda,[2] Atsushi Kumanogoh,[2,6,7] Hisashi Wada[1]

1 Department of Clinical Research in Tumor Immunology, Graduate School of Medicine, Osaka University, Osaka, Japan.

2 Department of Respiratory Medicine and Clinical Immunology, Graduate School of Medicine, Osaka University, Osaka, Japan.

3 Department of Thoracic Oncology, National Hospital Organization Osaka Toneyama Medical Center, Osaka, Japan.

4 Bioanalysis Department, Advanced Technology Center, Medical Solution Segment, LSI Medience Corporation, Tokyo, Japan.

5 Department of Respiratory Medicine, National Hospital Organization Osaka Toneyama Medical Center, Osaka, Japan.

6 Department of Immunopathology, Immunology Frontier Research Center, Osaka University, Osaka, Japan.

7 Integrated Frontier Research for Medical Science Division, Institute for Open and Transdisciplinary Research Initiatives (OTRI), Osaka University, Osaka, Japan.

*To whom correspondence should be addressed:

Kota Iwahori

Department of Clinical Research in Tumor Immunology, Graduate School of Medicine, Osaka University, 2-2 Yamadaoka, Suita, Osaka 565-0871, Japan.

E-mail address: iwahori@climm.med.osaka-u.ac.jp

Supplementary Table S1 Characteristics of severe adverse events.

Severe adverse events	
Pneumonitis	3
Colitis	1
Myositis	1
Liver dysfunction	1
Leukoencephalopathy	1
Ileus	1
Herpes zoster	1

Supplementary Fig. S1

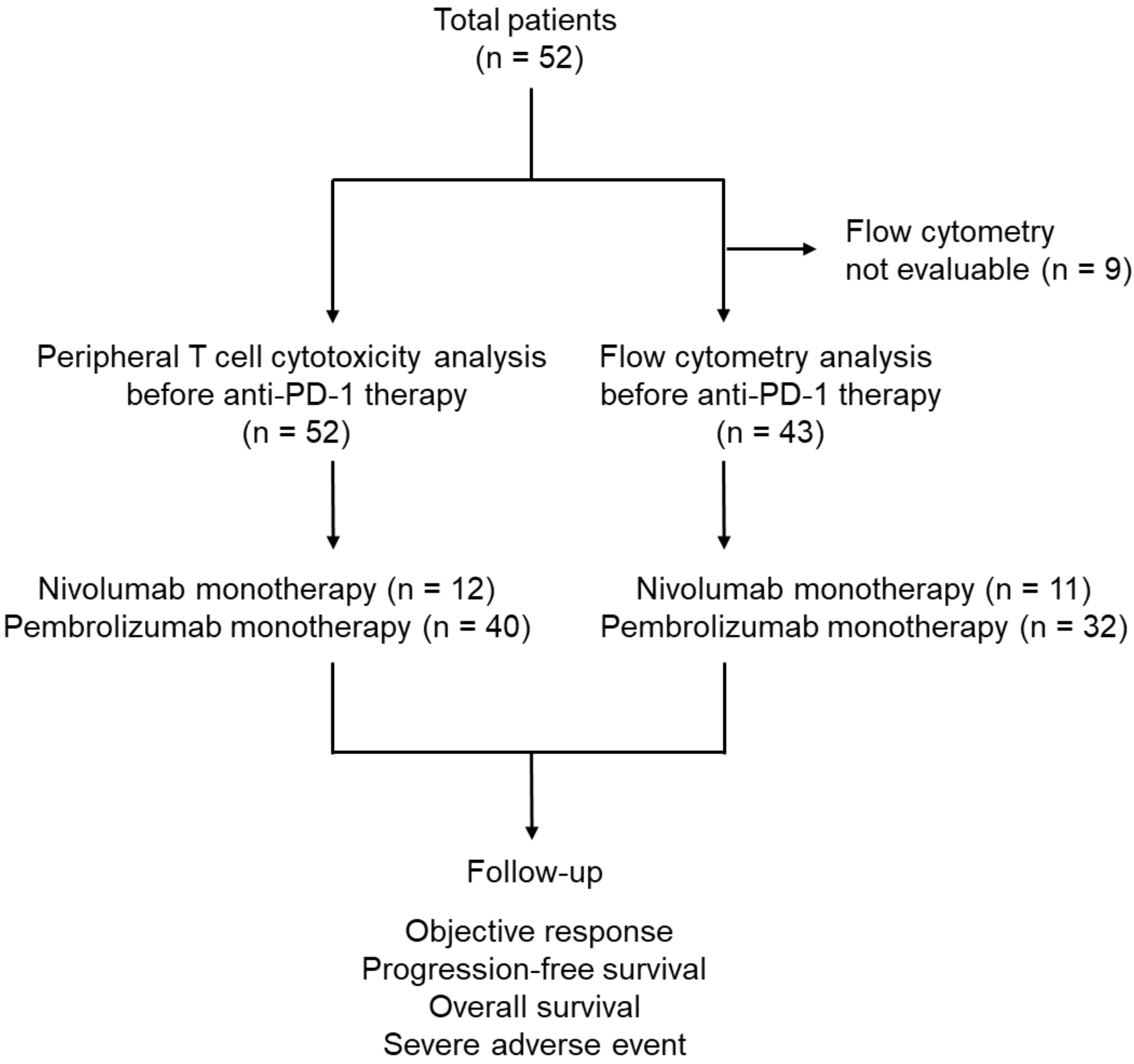

Supplementary Fig. S1   Flow diagram of patients and samples obtained.

Supplementary Fig. S2

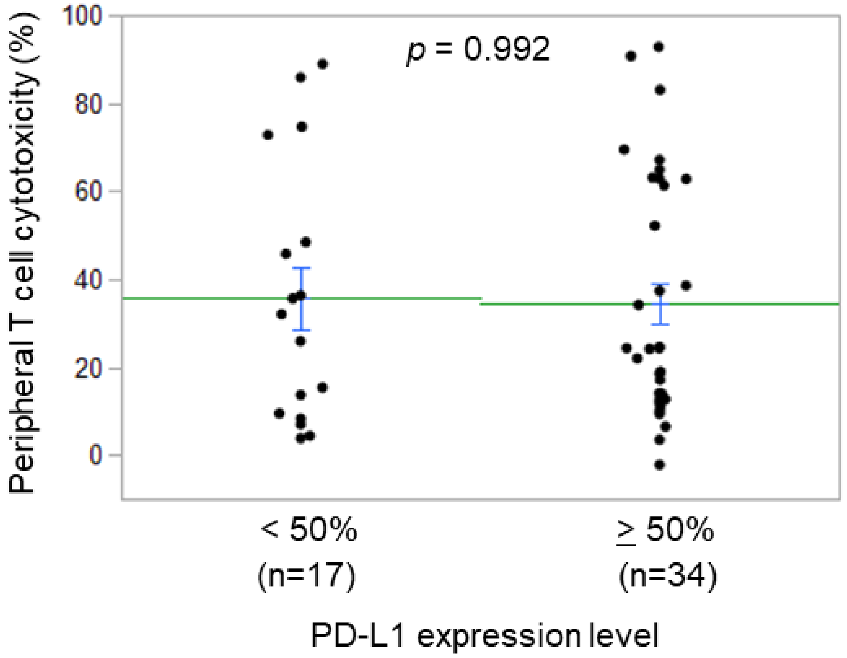

Supplementary Fig. S2 Comparison of peripheral T cell cytotoxicity according to PD-L1 expression levels.
Comparison of peripheral T cell cytotoxicity according to PD-L1 expression levels. Each dot represents one patient. Data represent the mean ± standard error of the mean (SEM). The significance of differences was assessed using the Mann-Whitney's U test.

Supplementary Fig. S3

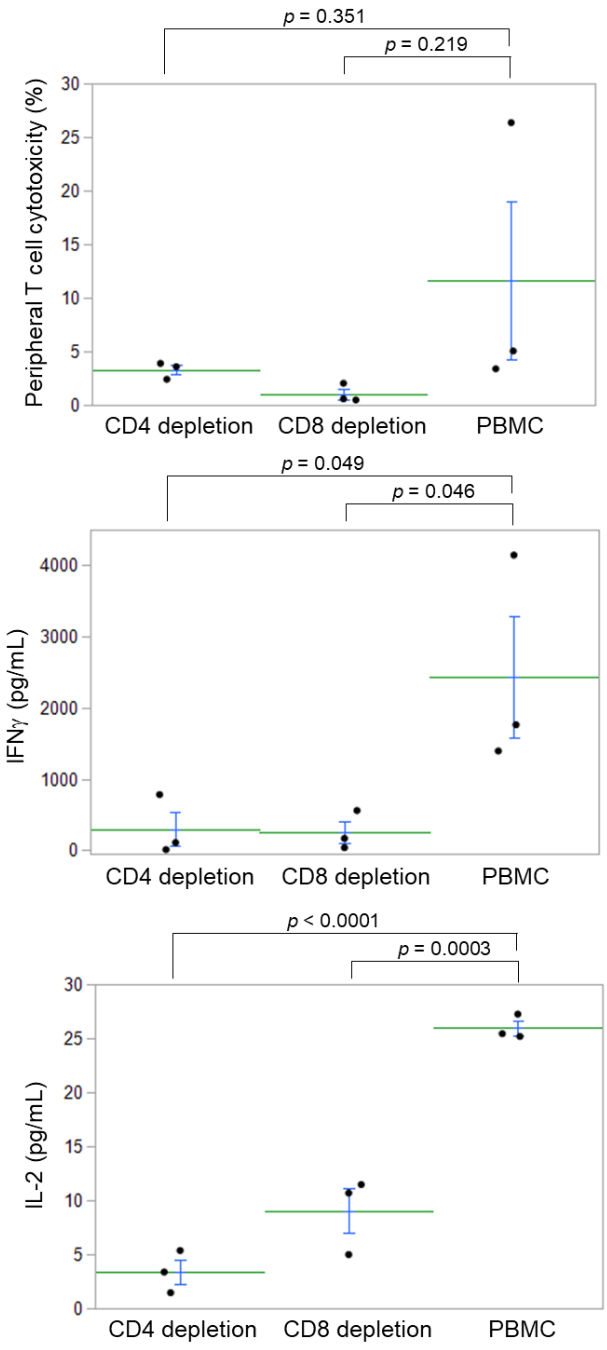

Supplementary Fig. S3 Peripheral T cell function without CD4+ or CD8+ T cells. Peripheral T cell cytotoxicity in healthy donors (n =3) was measured by a co-culture with PBMC or PBMC depleted of CD4+ or CD8+ T cells. IFNγ and IL-2 levels in co-culture supernatants were measured by ELISA (R&D Systems). The depletion of CD4+ or CD8+ T cells was achieved using CD4 or CD8 microbeads (Miltenyi Biotec). A one-way ANOVA with Dunnett's post hoc test was employed for multiple comparisons to compare differences with respective values for the control.

Supplementary Fig. S4

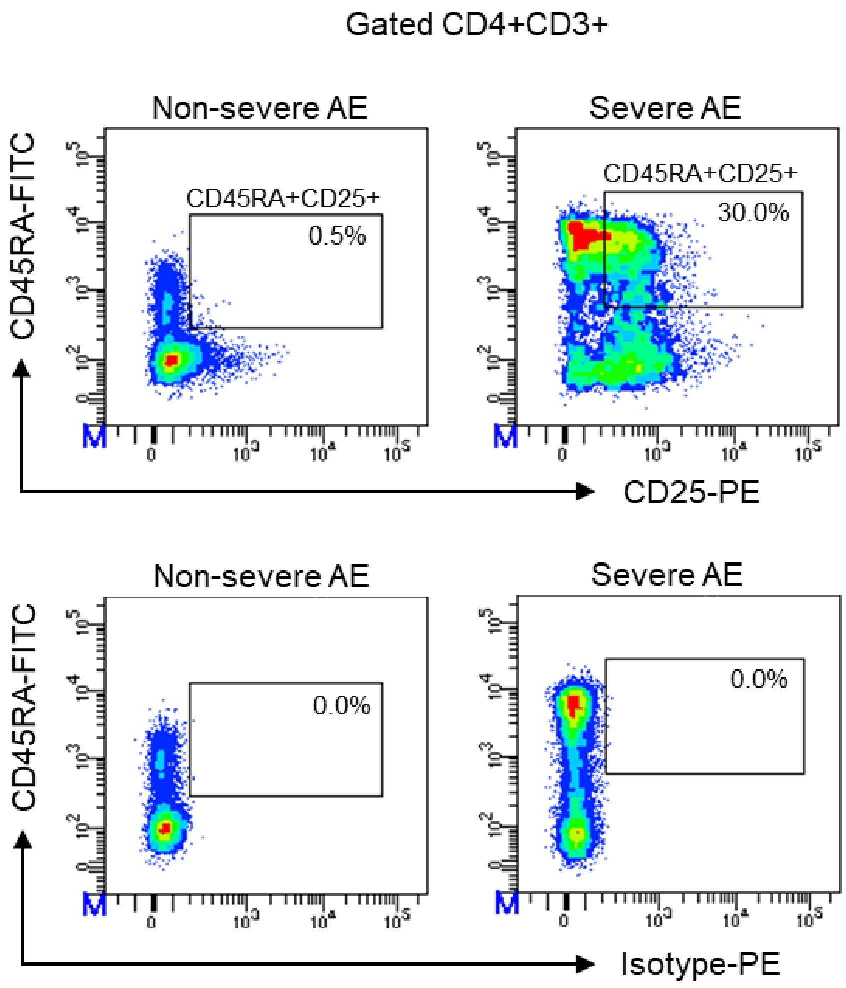

Supplementary Fig. S4 Two representative samples (non-severe AE and severe AE) showing CD45RA and CD25 positivity after gating on CD4+CD3+ PBMCs. AE, adverse event.

Supplementary Fig. S5

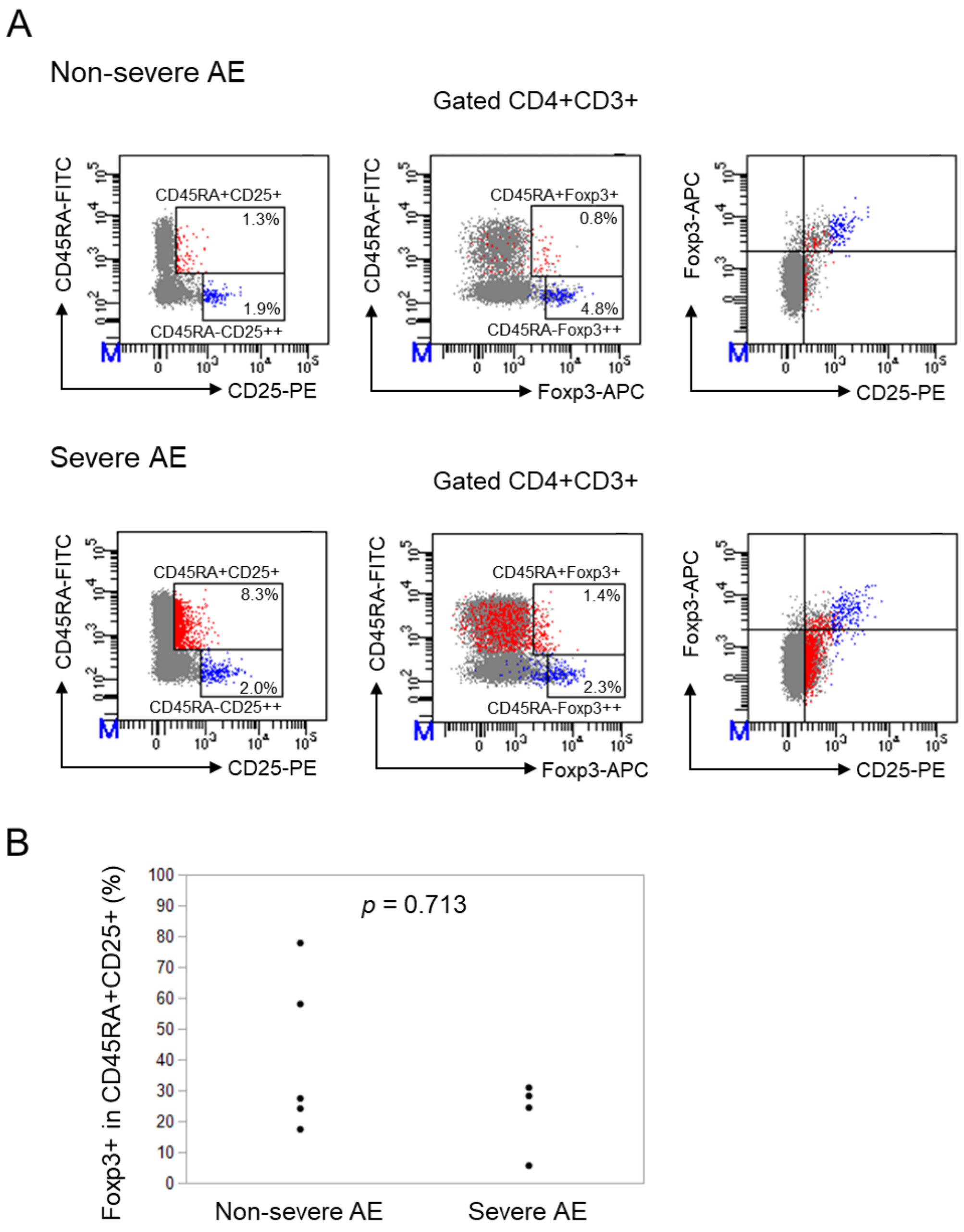

Supplementary Fig. S5 Foxp3 expression in CD4+CD45RA+CD25+ T cells. (A) Two representative samples (non-severe AE and severe AE) showing Foxp3 and CD25 positivity after gating on CD4+CD3+ PBMCs. Intracellular Foxp3 staining was performed by a Foxp3/Transcription Factor Staining Buffer Kit (Thermo Fisher Scientific) with an anti-Foxp3-APC antibody (clone PCH101, Thermo Fisher Scientific). Red dots represent CD4+CD45RA+CD25+ T cells. Blue dots represent CD4+CD45RA-CD25++ T cells. (B) The ratio of Foxp3+ in CD4+CD45RA+CD25+ T cells was compared between patients with non-severe AE (n = 5) and severe AE (n = 4). The significance of differences was assessed using the Mann-Whitney U test.

Supplementary Fig. S6

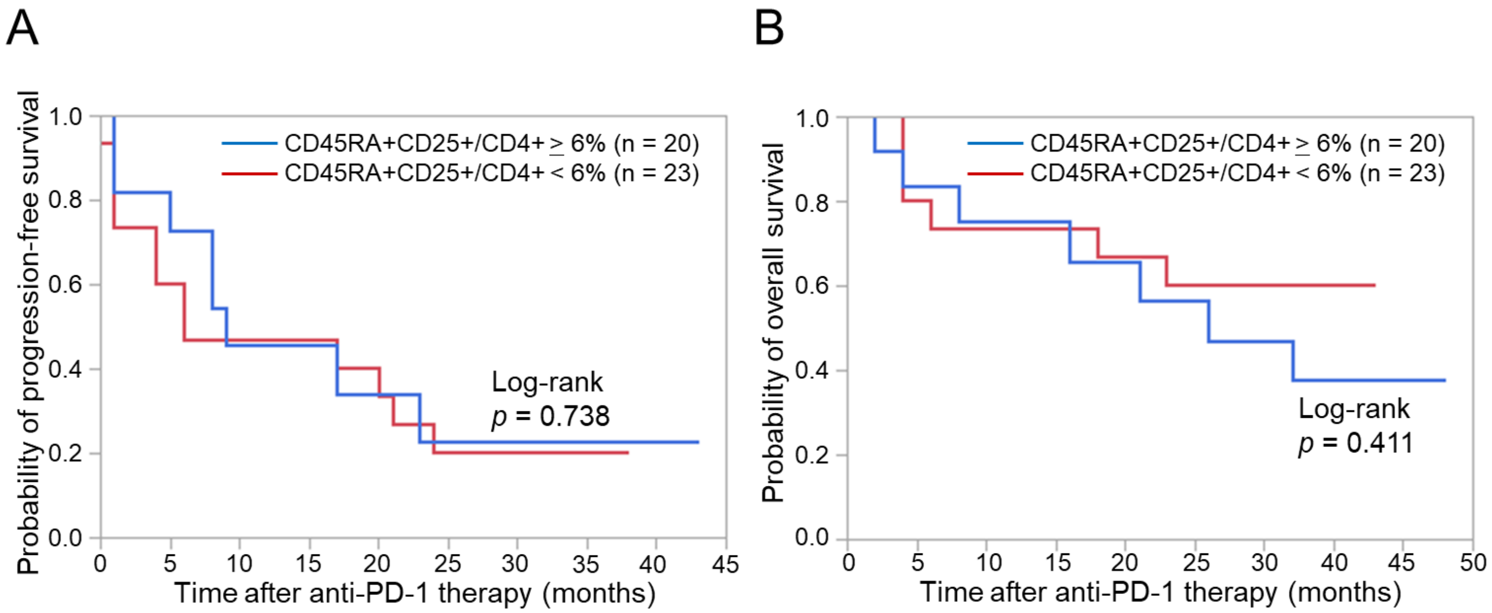

Supplementary Fig. S6 PFS and OS of patients with a PD-L1 tumor proportion score (TPS) ≥50% according to the peripheral CD45RA+CD25+/CD4+ T cell ratio. (A) Kaplan-Meier curves for PFS of patients with TPS ≥50% according to the CD45RA+CD25+/CD4+ T cell ratio. (B) Kaplan-Meier curves for the OS of patients with TPS ≥50% according to the CD45RA+CD25+/CD4+ T cell ratio. The significance of differences was assessed using the Log-rank test (A-B).

Supplementary Fig. S7

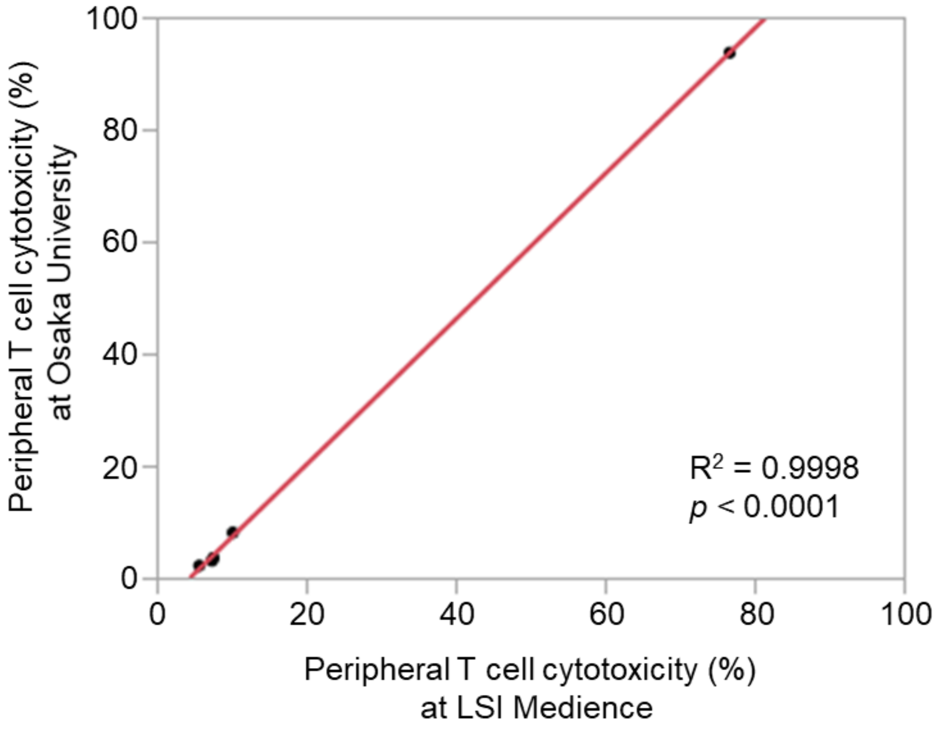

Supplementary Fig. S7 Comparison of peripheral T cell cytotoxicity measured at two institutes.

Fresh PBMCs ware analyzed at the laboratory of Osaka University and the same PBMCs were analyzed at the laboratory of LSI Medience Corporation on the next day after transportation from Osaka to Tokyo at room temperature. Each dot represents one donor (n = 5). Correlations between paired data were analyzed using Pearson's correlation coefficient.

Supplementary Fig. S8

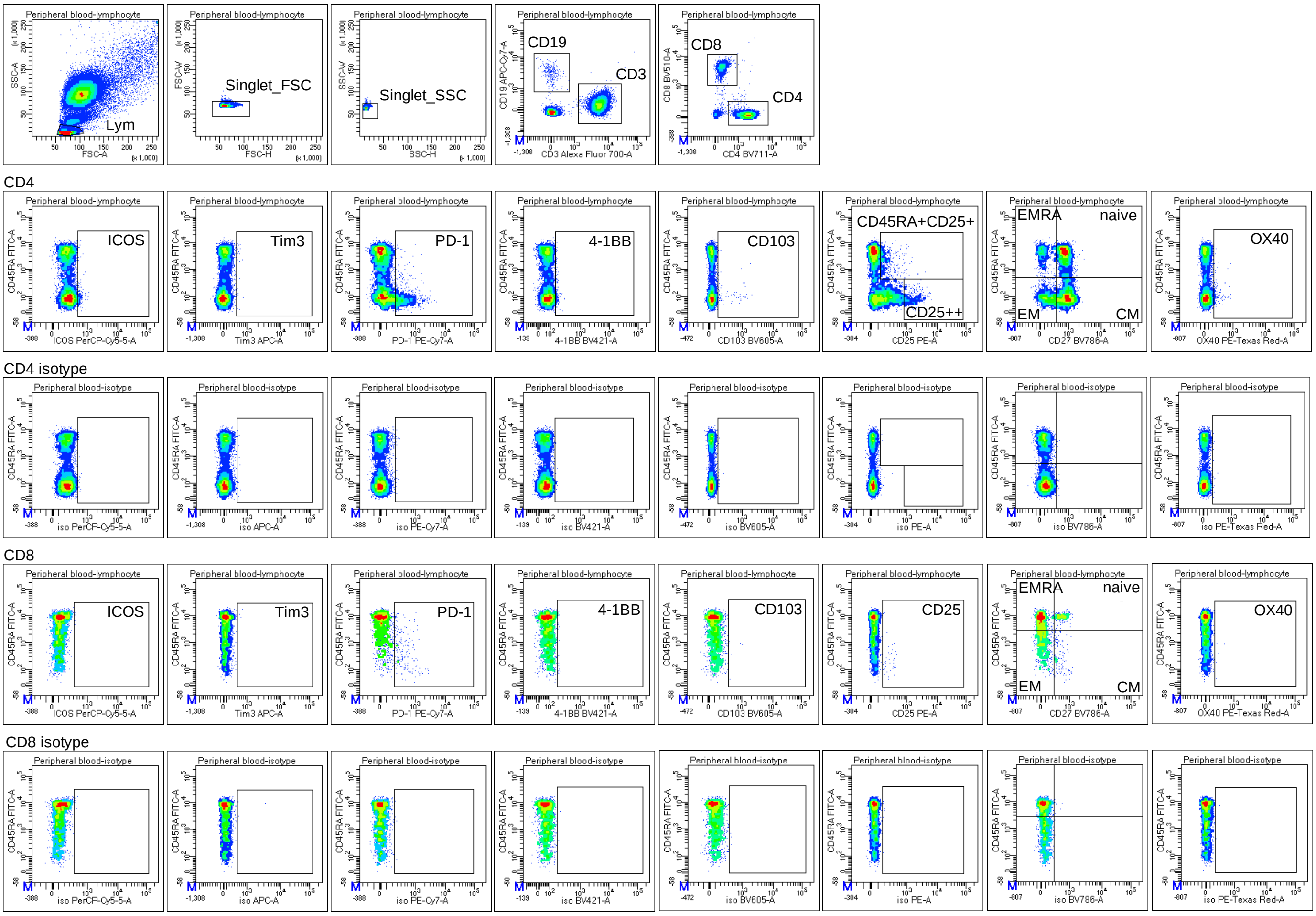

Supplementary Fig. S8 Gating strategy of the flow cytometric analysis.
The gating strategy of the flow cytometric analysis for peripheral blood was shown using BD LSRFortessa with FACSDiva software. CM, central memory; EM, effector memory; EMRA, effector memory re-expressing CD45RA.
